# The Use of Technology by the Rural-Dwelling Caregivers of People Living With Dementia to Support Caregiving: Qualitative Interview Study

**DOI:** 10.2196/77231

**Published:** 2025-09-24

**Authors:** Anna Jolliff, Sarah Boucher, Jordan R Hill, Kristen Allen-Watts, Miriam J Rodriguez, Christian Elliott, Matthew Zuraw, Nicole E Werner

**Affiliations:** 1 Center for Research and Innovation in Systems Safety Department of Anesthesiology Vanderbilt University Medical Center Nashville, TN United States; 2 Health & Wellness Design Indiana University School of Public Health - Bloomington Bloomington, IN United States; 3 The Division of General Internal Medicine and Population Science School of Medicine University of Birmingham Birmingham, AL United States; 4 CareVirtue Technologies, Inc San Diego, CA United States

**Keywords:** family caregivers, informal caregivers, rural populations, rural health, digital health, information technology, qualitative research

## Abstract

**Background:**

Family caregivers of people living with dementia who live in rural areas face challenges distinctly different from those experienced by their urban counterparts. Technology is a promising but underused method for delivering interventions to rural caregivers.

**Objective:**

This study aims to describe the technologies used by rural caregivers of people living with dementia, how these caregivers use technology to support caregiving, and the barriers and facilitators they encounter in using technology.

**Methods:**

We conducted online, semistructured interviews with rural caregivers of people living with dementia. The objective of the primary study was to understand how caregivers access support, including the types of support used, strategies used to find support, and any unmet support needs. This study was a secondary analysis focused exclusively on caregivers’ technology use. Summative, content, and thematic analyses were used to understand patterns of technology use and the associated facilitators.

**Results:**

A total of 19 caregivers were included in this study, of whom 14 (74%) were female. The average age was 66.4 (SD 10.1) years. The 5 most frequently endorsed technologies were phones for calling (n=19, 100%) and texting (n=14, 74%), websites (n=17, 89%), television and movies (n=15, 79%), and emails (n=13, 68%). Technology provided the following types of support: informational (n=19, 100%), emotional (n=13, 68%), instrumental (n=12, 63%), entertainment (n=10, 53%), safety (n=6, 32%), and caregiver personal health (n=4, 21%). Thematic analysis yielded 4 characteristics of caregivers that facilitated technology use, including access to technology, technological savviness, preference for technology, and having a technology broker. In addition, analyses yielded 4 characteristics of technology that facilitated technology use, including appeal, efficiency, ease of use, and trustworthiness.

**Conclusions:**

All rural caregivers in this study used technology to access caregiving support, which suggests that this population is prepared for remote interventions. Our findings can be used to help determine optimal delivery and content of these interventions and instruct interventionists on caregiver and technology characteristics that may facilitate uptake. Rural caregivers may be receptive to interventions delivered through texts, websites, and videos, particularly those that focus on meeting informational and instrumental needs.

## Introduction

### Background

In the United States, approximately 12 million Americans care for someone living with dementia, totaling 19 billion hours of care annually [1]. While this care is integral to the well-being of people living with dementia, it has consequences for family caregivers. Family caregivers face numerous social, emotional, physical, and financial challenges [1]. In rural areas, family caregivers contend with challenges different from those of their urban counterparts, such as difficulty finding transportation to medical appointments, lower health literacy, fewer available caregiving resources, and less awareness of existing resources [2]. It is imperative to develop interventions uniquely suited for and accessible by this population of rural caregivers.

One promising method of delivering interventions to rural caregivers is through technology. Recent studies on the use of information and communication technologies by older adult caregivers living in rural areas have demonstrated numerous benefits of technology use, including improved access to services, enhanced social support, and a reduced sense of isolation [3,4]. However, technology-based interventions for rural caregivers of people living with dementia are scarce. One recent review of 11 studies on health interventions for rural caregivers of people living with dementia found that less than half used technology and, of those that did use it, only 2 interventions leveraged technology that was more advanced than a phone call (one used online education and another video recordings) [5]. Similarly, a recent systematic review of 30 technology-based interventions for rural family caregivers of people living with dementia found that most interventions were delivered via the phone [6]. The extent to which other forms of technology can be leveraged to support caregiving by rural caregivers of people living with dementia is yet unknown.

Understanding current patterns of technology use is a necessary first step toward designing useful digital interventions. To date, there is little research describing the technology use patterns and preferences of rural caregivers of people living with dementia. It has been established that older adults’ use of technology is on the rise, with ownership of smartphones, tablets, smart televisions, home assistants, and wearables increasing among people aged between 60 and 69 years [7]. Furthermore, research suggests that older adults in rural areas can use technology to obtain support, including emotional, informational, and instrumental support [8-10]. However, technology use in rural areas may be threatened by the lack of access to high-speed internet and cost [7]. In addition, caregivers of people living with dementia may face barriers to technology use above and beyond those of other rural adults, including more demands on their time and less support in using technology [11].

In sum, there is reason to think that technology-based interventions could meet the many challenges faced by rural caregivers of people living with dementia. However, research is needed to understand viable technology-based methods of delivery as well as barriers to and facilitators of using technology to access support within this population.

### This Study

This study asked the following research questions:

1. What technologies do rural caregivers describe using to support caregiving?
2. For what supportive purposes do rural caregivers describe using technology?
3. What are the barriers and facilitators described by rural caregivers when using technology for support?

## Methods

### Study Design

We conducted remote, semistructured interviews with 19 rural caregivers of people living with dementia from November 2022 to January 2023. The objective of the primary study was to understand how caregivers access support, including the types of support used, strategies used to find support, and any unmet support needs. The development of the interview guide was informed by this study’s objective. An example question was, “Can you tell me what services and resources you know about in your community?” The interviewer would then probe to understand the process of finding out about these resources, including who helped the caregiver and any technologies used. Questions were initially drafted by the research team and then refined based on input from the study’s strategic advisory board. The strategic advisory board comprised 7 professionals who worked with caregivers of people living with Alzheimer disease or related dementias who lived in rural areas. Members were selected to ensure diverse representation in terms of geographic region, resource provision, roles, and expertise. This study was a secondary analysis that used qualitative methods to describe caregivers’ use of technology to access support.

### Ethical Considerations

Ethics approval for this study was granted by the Indiana University Bloomington Institutional Review Board. The board considered this study to involve minimal risk to human participants and thus granted expedited review (16214). A waiver of signed consent was approved. Participants received a study information sheet via email that outlined the risks and benefits of participation; then, they provided verbal consent to participate during a screening call. The study information sheet noted that the researchers might use participants’ interview data for secondary analyses without obtaining additional consent. Participants were encouraged to avoid the use of names or identifying information in interviews. When identifying information was shared, it was redacted from the transcript such that the final dataset was deidentified. Participants who completed the interview received a US $50 electronic gift card, which was emailed to them within 1 week of completing the interview.

### Setting and Participants

We used purposive and network sampling to recruit rural caregivers of people living with dementia in collaboration with community organizations serving rural areas, such as area agencies on aging, as well as in partnership with our strategic advisory board. Representatives from agencies and strategic advisors were invited to share the research opportunity electronically and in person. Recruitment contexts included digital newsletters and emails as well as organizations’ electronic and in-person support group meetings and education sessions. The inclusion criteria included being an unpaid caregiver of someone living with dementia, identifying their geographic area as rural, being aged at least 18 years, and having access to a phone to conduct the interview. Interested participants emailed or called the research team. A research team member scheduled the enrollment visit phone call and emailed the participant a copy of the study information sheet. During the enrollment call, participants were briefed on the study and invited to ask questions. They then provided verbal informed consent and scheduled the interview. All interviews took place over the phone or Zoom (Zoom Communications, Inc), according to the caregiver’s preference. Participants had no relationship with researchers before the study commencement. No participants dropped out after enrollment.

### Study Procedure

Interviews lasted for 60 to 90 minutes. Each interview was attended by a lead facilitator (AJ or NEW, both of whom identify as women) and a notetaker (JRH or MZ, who identify as a woman and a man, respectively). At the beginning of the interview, the interviewer and notetaker briefly shared with the participant their own professional and personal interest in the project. Participants completed an electronic survey in which they provided demographic details. All interviews were audio recorded and transcribed for analysis.

### Analysis

All analyses took place in NVivo (version 14; Lumivero). To answer research question 1, “What technologies do rural caregivers describe using to support caregiving?” we completed a summative analysis in which we documented each type of technology that caregivers used [12]. Transcripts were first reviewed line by line, and each passage describing technology use was extracted. These passages were then coded for the types of technology used. For example, if a caregiver described attending a support group over Zoom, it was coded that they used Zoom, and if they asked for help in a group message, it was coded that they used the text messaging app on their phone. Two coders (AJ and SB) coded a 20% subsample of transcripts to ensure consistency in coding. The remaining 14 transcripts were coded by either one of the coders.

To answer research question 2, “For what supportive purposes do rural caregivers describe using technology?” we performed a content analysis that combined deductive and inductive approaches [12]. As with the first research question, all passages in which technology use was described were extracted and coded. The initial codebook included 3 common types of support described in the caregiving literature: emotional, instrumental, and informational [13,14]. Two coders identified all passages in which caregivers described using technology for support and then coded these segments for the type of support sought. For example, if a participant described going online to find information about local resources for caregivers, this was coded as “informational support,” whereas if they texted a friend to request respite care, this was coded as “instrumental support.” Again, 2 coders (AJ and SB) coded a 20% subsample of transcripts to ensure consistency in coding. Coders made a note of any questions or challenges encountered during coding and discussed these in weekly coding meetings. Questions that could not be resolved between the two coders were brought to the whole research team for consensus-based discussion. Types of support that did not fit into the existing coding scheme were assigned a new code. The final codebook can be found in the Results section.

To answer research question 3, we used reflexive thematic analysis guided by the following research question: “What are the barriers and facilitators experienced by rural dementia caregivers when using technology for support?” We used this approach because we did not expect to identify a single “correct” interpretation of the data but rather an interpretation that was the product of coders’ perspectives [15]. Two coders (AJ and SB) separately read each transcript line by line and identified passages in which barriers to and facilitators of technology use seemed to have been described. Coders individually assigned a name to the preliminary barrier or facilitator. At weekly intervals, coders met to discuss the codes that they had created based on the passages they had identified. A codebook was created and iterated upon to reflect the coders’ evolving understanding of the data. After 50% of the transcripts had been coded, 3 additional members of the research team (NEW, MJR, and KA-W) coded a transcript each using the codebook in progress and discussed the extent to which they felt the codebook reflected their interpretation of the data. The goal of this discussion was not consensus but rather crystallization, that is, a multifaceted perspective on the data based on the reflections of multiple coders [16].

Data interpretation was shaped by the analysis team members’ perspectives. The analysis team included researchers with doctoral degrees in health education and promotion (KA-W), clinical neuropsychology (MJR), and human factors and applied cognition (NEW); a research coordinator with a master’s degree in counseling psychology (AJ); and an undergraduate student studying community health (SB). Data analysts’ training inevitably shaped our impressions of the data [15]. This is particularly true for research questions 2 and 3, for which data analysis required more interpretation. Our recognition and categorization of support seeking and technology use as well as our understanding of barriers to and facilitators of use were interpretations based on our professional and, to some extent, personal experiences. Interpretation was further required because this study was a secondary analysis; in the interviews, we did not explicitly ask all the research questions that we later set out to answer through secondary analysis.

## Results

### Demographic Information

Of the 19 caregivers, 14 (74%) were female. Caregivers’ average age was 66.4 (SD 10.1) years. Within the sample, 11 (58%) caregivers cared for a partner, 6 (32%) cared for a parent or parent-in-law, 1 (5%) cared for a child, and 1 (5%) cared for a friend. Table 1 presents complete participant demographic information.

**Table 1:** Demographic information for a sample of rural caregivers of people living with dementia (N=19).

Characteristic		Values
Age (y), mean (SD)		66.4 (10.1)
**Gender^a^, n (%)**		
	Man	5 (26)
	Woman	14 (74)
**Race and ethnicity^a^, n (%)**		
	American Indian or Alaska Native	1 (5)
	Non-Hispanic White	18 (95)
**State, n (%)**		
	South Dakota	13 (68)
	Wisconsin	3 (16)
	California	2 (11)
	Washington	1 (5)
**Education, n (%)**		
	Graduate or professional degree	6 (32)
	Bachelor’s or associate’s degree	10 (53)
	Some college or no degree	2 (11)
	High school diploma or GED^b^	1 (5)
**Employment, n (%)**		
	Working full time	4 (21)
	Working part time	1 (5)
	Retired	9 (47)
	Other	5 (26)
**Income (US $), n (%)**		
	<25,000	3 (16)
	25,000-49,999	2 (11)
	50,000-74,999	8 (42)
	75,000-99,999	1 (5)
	100,000-149,999	3 (16)
	Prefer not to say	2 (11)

^a^Only 2 response options were selected by participants.

^b^GED: general educational development.

### Technologies Used to Support Caregiving

The 5 most frequently mentioned technologies by the 19 caregivers were phones for calling (n=19, 100%) and texting (n=14, 74%), websites (n=17, 89%), television and movies (n=15, 79%), and emails (n=13, 68%). Table 2 presents a comprehensive list of the technologies mentioned by 1 or more caregivers.

**Table 2:** Technologies used by rural caregivers of people living with dementia to support caregiving (N=19).

Type of technology			Caregivers, n (%)
**Phone**			
	Call app	19 (100)	
	Text app (individual or group)	14 (74)	
	Other app	3 (16)	
Website			17 (89)
Television and movies			15 (79)
Email			13 (68)
**Videoconferencing software**			
	Zoom or webinar	11 (58)	
	Telehealth	4 (21)	
	View clicks (video calling)	1 (5)	
Social media (including YouTube)			6 (32)
Audio (music and audiobook)			4 (21)
**Surveillance system**			
	Camera	4 (21)	
	Door alarm	2 (11)	
	Motion-detecting lights	1 (5)	
	Sensor on the walker	1 (5)	
	Speaker	1 (5)	
	Call button	1 (5)	
Smart watch			3 (16)
Videogames			3 (16)
Tablet			2 (11)
Personal assistant			1 (5)
Computer application			1 (5)
Digital pill dispenser			1 (5)

### Purposes of Technology Use

In order of frequency, technology use was described for the following purposes by the 19 caregivers: informational support (n=19, 100%), emotional support (n=13, 68%), instrumental support (n=12, 63%), entertainment (n=10, 53%), safety (n=6, 32%), and caregiver personal health (n=4, 21%). The purposes of entertainment, safety, and caregiver personal health were not part of our initial coding framework but were arrived at inductively. Table 3 presents examples of each type of support and the percentage of caregivers who described using technology for that type of support.

**Table 3:** Types of technology-based support obtained by rural caregivers of people living with dementia (N=19).

Type of support	Definition	Examples from transcripts	Caregivers, n (%)
Informational support	Technology to facilitate knowledge sharing	Researching aging or dementia online, participating in live webinars about dementia care, receiving email newsletters from area agencies on aging, and requesting information in online support groups	19 (100)
Emotional support	Technology to facilitate the expression of empathy or caring	Commiserating in support groups, calling church members with prayer requests, expressions of caring from Facebook friends, and texting other caregivers to ask how they are doing	13 (68)
Instrumental support	Technology to facilitate the provision of tangible aid	Texting family or friends to request respite care, going online to find an attorney, calling a friend to help when the care recipient has had a fall, calling government agencies to request services, and watching instructional YouTube videos	12 (63)
Entertainment	Technology to amuse or divert the care recipient	Recording care recipient’s favorite television shows, allowing friends and family to video call care recipient, and care recipient playing video games alone or with others	10 (53)
Safety	Technology for the protection of the care recipient	Sensor to alert caregiver when care recipient gets out of bed at night, smartwatch to enable care recipient to easily call recipient, cameras to monitor care recipient when away from home, door alarms to know if care recipient is leaving, and checking on care recipient’s safety over text	6 (32)
Caregiver personal health	Technology to facilitate the physical or emotional well-being of the caregiver	Listening to a podcast or audiobook while driving the care recipient, caregiver monitoring their own sleep with a smartwatch, and taking an online weight loss class	4 (21)

### Barriers to and Facilitators of Technology Use

Through reflexive thematic analysis, we perceived 8 factors that could serve as a facilitator of or a barrier to technology use for rural caregivers. We divided these themes into 2 imperfect categories: characteristics of caregivers and characteristics of technology. We perceived the following characteristics of caregivers: access to technology, technological savviness, preference for technology, and access to a technology broker. Furthermore, we perceived the following characteristics of technology: appeal, efficiency, ease of use, and trustworthiness. These categories are imperfect because, for example, trustworthy technology could also reflect a trusting caregiver, while a caregiver’s ability to access technology could reflect an accessible technology. However, these categories were valuable in that they facilitated the organization of ideas. Multimedia Appendix 1 presents definitions of each thematic barrier or facilitator and example quotations from participants.

## Discussion

### Principal Findings

In this study, we sought to explore the technology use of rural caregivers of people living with dementia, including the technologies they described using, the supportive purposes for which they used technology, and any factors that seemed to serve as a barrier to or facilitator of use. Our findings suggest that rural caregivers’ technology use goes well beyond making phone calls and watching television to include texting, visiting websites, emailing, videoconferencing, social media use, and more. Every caregiver we interviewed used technology for informational support, and more than half of them used technology for emotional support (13/19, 68%), instrumental support (12/19, 63%), and entertainment (10/19, 53%). Thematic analyses yielded 8 factors that, when present, seemed to facilitate technology use. Taken together, these findings can be used to help determine the optimal delivery and content of technology-based interventions for the population of rural caregivers of people living with dementia.

In combination with existing research, our findings suggest that the current array of technology-based caregiver interventions may underestimate this population’s level of technological engagement and ingenuity [17]. Currently, most technology-based interventions for caregivers, including rural caregivers, use phone calls as the technological medium [18]. In this study, while it was true that all 19 rural caregivers used phone calls, many also used websites (n=17, 89%), group or individual texting (n=14, 74%), emails (n=13, 68%), and videoconferencing (n=11, 58%). There are benefits to these technologies as mediums for intervention delivery. Unlike phone call–based interventions, web- and email-based interventions can be self-paced and contain more information than can be delivered via phone [18]. Web-based interventions can also be made more accessible than phone-based interventions by adhering to web content accessibility guidelines, which offer instruction on presenting content in multiple, customizable formats [19]. In text messaging–based interventions, contact is often more frequent than in phone call–based interventions; therefore, participants may feel more continuously supported [20]. Furthermore, the text message “thread” automatically creates a record of all resources shared with the caregiver [20]. Finally, interventions delivered via videoconferencing have the power to facilitate deeper interpersonal connections among caregivers who would otherwise be geographically isolated [21,22]. We therefore advocate for the development and testing of new technology-based interventions for rural caregivers of people living with dementia. We further advocate for using technologies that are already familiar to many caregivers (eg, websites, text messaging, and online support groups) to disseminate information about more advanced technologies with unique affordances, such as caregiver-focused communities on TikTok [23] and virtual reality experiences [24] that educate dementia caregivers.

Previous research has reviewed the content of interventions for caregivers and has found that the content predominantly focuses on psychosocial outcomes, such as burden and self-efficacy [5]. However, there is a lack of interventions for rural caregivers that focus on providing the instrumental aid that is much needed in rural areas, such as addressing the effects of poverty and increasing access to health care services (eg, medical specialties and home nursing) [5]. In this study, 63% (12/19) of the caregivers used technology, such as web, text, email, and videoconferencing, to find instrumental assistance. Furthermore, all caregivers in this study used technology to find information. However, interventions for caregivers of people living with dementia that focus on education and information sharing are outnumbered by those focusing on psychosocial outcomes [25]. Our results support the development of technology-based interventions that provide instrumental and informational aid to rural caregivers of people living with dementia, with objectives such as finding long-term care; accessing local, state, and federal caregiver programs; and obtaining professional assistance with financial, legal, and health care planning that may be scant in rural areas [26]. It is also possible that smartphone apps and websites that are already commercially or freely available could meet caregivers’ instrumental and informational needs. However, the sheer number of resources may overwhelm busy caregivers [27]. Our findings suggest that caregivers would benefit from clinical, community, and technological brokers (eg, medical professionals, older adult service providers, and user experience designers) who can screen existing resources on the metrics that mattered to caregivers in this study, such as trustworthiness and the ease of use.

Many of the facilitators of and barriers to technology use yielded by our analyses overlap with existing research on technology use among older adults. With both rural caregivers and the larger population of older adults, facilitative characteristics include having access to technology (including being able to afford it), technological savviness or knowledge, interest in technology, and the presence of “brokers” (family and friends) who can guide technology use [7,17,28,29]. For both groups, characteristics that act as barriers to technology use include the technology being untrustworthy and difficult to use [7]. This suggests that the existing efforts to improve technology design for older adults may generalize to rural caregivers [30]. However, to reach rural older adults, efforts to help older adults get online must be improved [31]. In our sample, all participants had access to home internet; however, national estimates of rural Americans’ access to broadband internet at home range from 72% to 86% [32,33]. Interest in technology-based caregiving interventions is likely to increase as individuals from Generation X and Generation Y become caregivers, given that their youngest members (aged 45 and 29 y, respectively, as of 2025) are either digital natives themselves or report high engagement with technology [34].

### Limitations

This study had some limitations. Although our sample was representative of dementia caregivers in that it was predominantly composed of women [35], it also disproportionately consisted of White individuals. Therefore, our results lack the critical perspective of caregivers in rural areas from other racial and ethnic groups. Second, in this study, we adopted an inclusive definition of rurality, considering caregivers to be rural if they self-identified as such. However, there are many distinct definitions of rurality; for example, in the United States, the US Census Bureau, the Centers for Disease Control and Prevention, and the United States Department of Agriculture all define rurality differently [36]. While we do not believe our operationalization of rurality to be a weakness of this study, it is important to keep it in mind while interpreting results [36]. Third, participating in a phone- or web-based interview was a condition of participation. This biased our sample toward caregivers who were more technologically connected. Future research should expand the in-person recruitment contexts to include more spaces in which rural caregivers may be found, such as religious organizations, medical offices, and grocery stores, and data collection contexts to include homes, libraries, and community centers. Finally, because this was a secondary analysis, our data collection procedure was not designed to understand exactly which technologies caregivers used or the breadth of their technology use. Therefore, participants may have had broader experience with technology than is reflected in our findings. Future quantitative research should adopt a more comprehensive measure of technology use and distribute it to larger samples of caregivers to further elucidate rural caregiver technology use.

### Conclusions

Our findings underscore the need for interventions that leverage technologies, such as websites, text messaging, and videoconferencing, to support rural caregivers. Caregivers in our study already used these technologies to obtain many types of support, suggesting that technology-enabled interventions targeting common areas of need for rural caregivers would be well received by this population. To further understand the technology use patterns and preferences of rural caregivers, future research must prioritize in-person or otherwise offline strategies for recruiting and engaging these caregivers. The resulting interventions may be as varied and nuanced in their deployment of technology and support as rural caregivers are themselves.

## Appendix

Multimedia Appendix 1 Barriers to and facilitators of technology use among rural caregivers of people living with dementia.

## Data Availability

The datasets generated or analyzed during this study are not publicly available due to the sensitivity of the data but are available from the corresponding author on reasonable request.
