# Supplementary material for: The Use of Technology by the Rural-Dwelling Caregivers of People Living With Dementia to Support Caregiving: Qualitative Interview Study
Source: JMIR Form Res. 2025 Sep 24;9:e77231. doi: 10.2196/77231 (PMC12508661; doi:10.2196/77231)
Supplement: Multimedia Appendix 1 [file formative_v9i1e77231_app1.docx]

**Multimedia Appendix**

**Table.** Barriers to and facilitators of technology use among rural caregivers of people living with dementia.

| Theme | | Definition | Barrier example | Facilitator example |
| --- | --- | --- | --- | --- |
| **Characteristics of caregivers** | | | | |
|  | Access to technology | Participant describes having or lacking access to technology (eg, computer and internet), including for reasons of cost or strength of internet connection. | - “Even though it’s a rural state and rural area, yeah, Internet is very good. The main concern is that one, many folks of my age do not have computers. They may have access to the Internet, but they may not be able to afford the Internet.” [P107] | “There’s a resource room at that nursing home…. I’ve never used it because I do have access to the Internet, and I don’t have to go to the library for stuff.” [P112] |
|  | Technological savviness | Participant describes being skilled or unskilled and comfortable or uncomfortable with technology. | - “Our cable company decided to make the remotes really confusing, so that’s not good for seniors when you can’t turn the TV on. You know, it’s like seniors don’t need Netflix and Prime and Hulu and all that.” [P117] | “I worked in technology for 20 years, so it’s second nature to me to think about tech as a solution to problems, yeah.” [P115] |
|  | Preference for technology | Participant describes own or others’ attitude toward technology; participant describes an inherent preference for using digital versus analog tools. | - “The majority of people [at support group], dare I say it, are little old ladies who are not really inclined to use technology. So the virtual ones just didn’t work very well…. I’m very comfortable with technology…. You know, face to face is better.” [P106] | “If you want to care for someone, you’re probably going to want to use technology to do it…. One of my big searches was how do I use technology to help my parents stay safe and help me keep them safe and keep my stress down?” [P103] |
|  | Having a technology broker | Participant describes another person as facilitating their technology use, or participant describes having no one available to help them with technology. | - “I’m not on Zoom, I have problems with it. I’m challenged in that way…. To do it on myself, by myself, I will hit the wrong button, and it doesn’t work if you don’t do things exactly the way it wants.” [P107] | “Our son lives across the driveway.… And he’s a computer guy. That’s what he does for a living. So he, you know, if we’re having trouble ever, he’ll tell me what to do, basically.” [118] |
| **Characteristics of technology** | | | | |
|  | Appeal | Participant describes some aspect of technology that makes it more or less visually appealing or attractive. | - Caregiver: “I don’t know why the state websites have to be so difficult to navigate. I think it should look cheerful…like it can look official but not be, oh, what’s the right word?” - Interviewer: “Like, sterile?” - Caregiver: “Yeah, that’s a good word.” [P108] | “There are a number of apps out there that are really good for dementia training. [The care recipient] looks at the little logo for the app, and the appearance of the logo will tell her whether she wants to do it or not.” [P114] |
|  | Efficiency | Participant describes the amount of time taken to use technology as a barrier or facilitator. | - “And the folks that attended, we found that, you know, they would try that [state organization] and just stop because it was so frustrating to try to get anywhere to call. The wait…. You know, it’s not, call that 1-800 number and get help…. Caregivers in [caregiver’s home state] qualify for services as a caregiver. And I haven’t done that either because, number one, I don’t have time.” [P108] | “If they switch [the support group] to all in person, I’m going to be kind of left out, because they are way down in [county]…. So it would take me an hour to get there. Yeah, it’s a long drive. But the Zoom is wonderful, so so far, it’s fine for me.” [P104] |
|  | Ease of use | Participant describes being able to find the information that they need using the technology they have. | “I didn’t like the sales workers that I ended up finding online. You are supposed to go through them instead of the administrator to find your place…. You talk to the salesman, and you can’t talk to the administrator of the place yourself, or they don’t want you to.” [P113] | “They do have a Facebook page, and they post a lot of good links to articles. Usually, if I’m having a bad day or thinking I’m the only one going through something, you know, or if she is experiencing something I think, this is just out of the ordinary, they’ll post something about it or have a link to a resource. And we’ll go through their Facebook page and click on there and kind of read up on it.” [P112] |
|  | Trustworthiness | Participant describes technology as earning their trust or distrust; participant says that the source of technology-based information is the reason they used or did not use it. | - “Resources like Alzheimer’s Association I totally trust and understand, and they’re very specific in how they conduct, where they get their information…. I don’t always trust Dr. Google, okay, or certain search engines or certain, like fraud is a big thing…. So that’s kind of why I stay close to pretty solid research.” [P116] | “If it’s something, you know, something that I feel like I can trust, then that’s a good resource…if [the URL is] something I recognize or it doesn’t look like it’s, you know, just some Joe Shmoe guy that's decided he’s got this miracle cure for Alzheimer’s….” [P1] |
